# The agreement between jugular bulb and cerebrospinal fluid lactate levels in patients with out-of-hospital cardiac arrest

**DOI:** 10.1038/s41598-024-59986-5

**Published:** 2024-04-22

**Authors:** Jung Soo Park, Yeonho You, Changshin Kang, Wonjoon Jeong, Hong Joon Ahn, Jin Hong Min, Yong Nam In, So Young Jeon

**Affiliations:** 1 Department of Emergency Medicine, Chungnam National University Hospital, 266 Munhwa-ro, Jung-gu, Daejeon, 35015 Republic of Korea; 2 Department of Emergency Medicine, College of Medicine, Chungnam National University School of Medicine, Daejeon, Republic of Korea; 3 Department of Emergency Medicine, Chungnam National University Sejong Hospital, Sejong, Republic of Korea

**Keywords:** Neuroscience, Medical research

## Abstract

We investigated the agreement between the jugular bulb (JB) and cerebrospinal fluid (CSF) lactate levels. The study was conducted from July 2021 to June 2023 as a prospective observational cohort study at a single center. The right jugular vein was accessed, and the placement of JB catheter tip was confirmed using lateral cervical spine X-ray. A lumbar catheter was inserted between the 3rd and 4th lumbar spine of the patient. Lactate levels were measured immediately, 24 h, 48 h, and 72 h after ROSC. In patients with a good neurological prognosis, kappa between JB and CSF lactate levels measured immediately, at 24 h, 48 h, and 72 h after ROSC were 0.08, 0.36, 0.14, − 0.05 (*p* = 0.65, 0.06, 0.48, and 0.75, respectively). However, in patients with a poor neurological prognosis, kappa between JB and CSF lactate levels measured immediately, at 24 h, 48 h, and 72 h after ROSC were 0.38, 0.21, 0.22, 0.12 (*p* = 0.001, 0.04, 0.04, and 0.27, respectively). This study demonstrated that JB lactate levels exhibited significant agreement with arterial lactate levels, compared to CSF lactate levels. Therefore, this should be considered when using JB lactate to monitor cerebral metabolism.

## Introduction

Cerebral ischemia/reperfusion injury following cardiac arrest (CA) is associated with several processes, including cell energy depletion, ionic pump failure, glutamate release, intracellular calcium influx, and release of reactive oxygen species and free radicals^1–4^. These processes lead to impaired cerebral oxygen and glucose metabolism, resulting in mitochondrial dysfunction, disruption of the blood–brain barrier (BBB), and neuronal cell death. Ultimately, this adversely affects the neurological prognosis of patients with out-of-hospital cardiac arrest (OHCA). During this series of events, lactate levels increase, which can be useful in predicting the prognosis of patients with OHCA. Specifically, cerebrospinal fluid (CSF) lactate measured 24 h after return of spontaneous circulation (ROSC) has been shown to have a better predictive performance for neurological prognosis than serum lactate levels^5^.

A previous study employed steroids as potential treatments for cerebral metabolism derangement; however, they have not been reported to significantly improve long-term neurological prognosis^6^. Nonetheless, as cerebral metabolism varies depending on the severity of OHCA, it is essential for future studies on cerebral metabolism derangement treatments to distinguish between cerebral ischemia and mitochondrial dysfunction based on the severity of OHCA^7^.

Jugular bulb (JB) microdialysis is a useful method for monitoring cerebral metabolism in patients with OHCA, reflecting brain damage^8^. However, one study has reported that CSF lactate, which reflects the anaerobic metabolism of cerebral glycolysis, is unrelated to serum lactate levels. Additionally, lactate levels measured in the internal jugular vein are similar to arterial lactate levels^9,10^. However, this study was conducted in animals, and it remains unclear which specific part of the internal jugular vein was used for measuring serum lactate.

Therefore, in this secondary analysis of the previous study^7^, we aimed to investigate the agreement between JB lactate levels obtained through a catheter located in JB and CSF lactate levels obtained through a lumbar catheter.

## Materials and methods

### Ethical approval and consent

This study was approved by Chungnam National University Hospital institutional Review Board (CNUH IRB 201907033003). All procedures and protocols were conducted in accordance with the Declaration of Helsinki and the International Conference of Harmonization and Good Clinical Practice guidelines. Written informed consent and approval for the donation of human materials and research on human medical ethics were obtained from the patient’s next of kin.

### Study design and patients

This prospective observational cohort study was conducted from July 2021 to June 2023 at a single center and targeted adult patients with OHCA who underwent target temperature management (TTM) with a Glasgow Coma Scale (GCS) score of 8 or lower immediately after ROSC. The neurological prognosis of the patients was evaluated 6 months after ROSC via face-to-face interviews or structured telephone interviews using the Glasgow–Pittsburgh cerebral performance category (CPC) scale^11^. Good neurological prognosis was defined as CPC 1 and 2, whereas poor neurological prognosis was defined as CPC 3–5. Face-to-face or structured telephone interviews were conducted by an emergency physician who understood the study protocol. The exclusion criteria were as follows: (1) CA due to trauma; (2) TTM discontinued due to hemodynamic instability; (3) TTM not performed due to cerebral hemorrhage, active bleeding, poor neurologic status prior to CA, or known terminal illness; (4) severe cerebral edema, obliteration of the basal cisterns, or an intracranial mass on brain computed tomography; (5) ineligibility for lumbar puncture due to antiplatelet therapy, anticoagulation therapy, coagulopathy, platelet count less than 40,000/mL, or international normalized ratio > 1.5^12^; (6) Extracorporeal membrane oxygenation provided; and (7) consent was not granted by the next of kin for this study. The following data were recorded: age, sex, presence of a witness at the time of the collapse, cardiopulmonary resuscitation (CPR) administered by the bystander, cardiac rhythm during the first monitoring, etiology of CA, time from collapse to CPR (no-flow time), time from CPR to ROSC (low-flow time), serum neuron specific enolase measured at 72 h after ROSC, Charlson comorbidity index, GCS scores immediately after ROSC, and CPC 6 months after ROSC. Contrast-enhanced magnetic resonance imaging was performed using a 3T scanner (Achieva3T, Philips Medical System, Andover, Netherlands) between 72 and 96 h after ROSC to evaluate the presence of BBB disruption.

### TTM protocol

TTM was conducted using cooling devices (Arctic Sun^®^ Energy Transfer Pads™, Medivance Corp., Louisville, KY), and core body temperature was monitored using esophageal and bladder thermometers. In cases where the cause of CA was of cardiac origin, and the patients exhibited a shockable rhythm, we maintained a target temperature of 36 °C for 24 h. For all other patients, we maintained a target temperature of 33 °C for 24 h. Afterward, patients were rewarmed at a rate of 0.25 °C/h up to 37 °C and then maintained at 37 °C for 72 h before discontinuing TTM.

### Measurement of serum lactate obtained from the jugular bulb and CSF lactate

**Figure 1:**
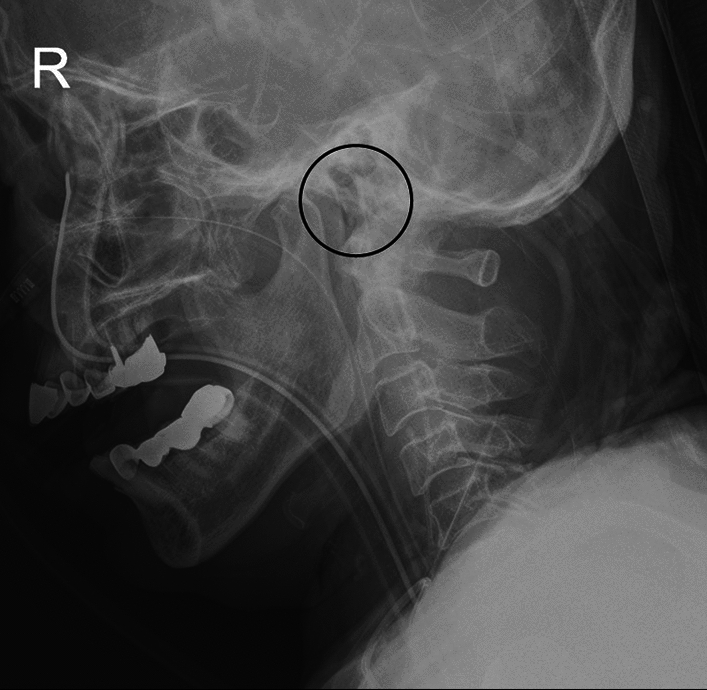
The placement of the catheter tip confirmed using lateral cervical spine X-ray. Lateral cervical spine X-ray documented a correct positioning of the jugular bulb catheter tip (black circle).

The right jugular vein was accessed through the retrograde insertion of a 22 G intravenous catheter (130 mm), with the tip placed in JB under ultrasound guidance. According to a previous study^8^, the placement of the JB catheter tip corresponds to the anatomical landmark at the level of the mastoid. The placement was confirmed using lateral cervical spine radiography (Fig. 1). A lumbar catheter was inserted using a Hermetic™ lumbar accessory kit (Integra Neurosciences, Plainsboro, NJ) between the 3rd and 4th lumbar spine of the patient, who was lying in the lateral decubitus position with the hip and knee flexed. Arterial samples were collected using a radial arterial catheter. Samples obtained from the arterial, JB, and lumbar catheters were centrifuged at 3000 rpm for 10 min, and the supernatants were immediately analysed. Lactate levels were measured immediately, 24 h, 48 h, and 72 h after ROSC.

### Statistical analysis

We planned to enrol 40 patients in accordance with the inclusion and exclusion criteria, as there were no previous studies reporting agreement between JB and CSF lactate levels in patients with OHCA. Continuous variables are reported as medians with interquartile ranges or means and standard deviations, depending on the normal distribution. Categorical variables are reported as frequencies and percentages. Comparisons between the arterial, JB, and CSF lactate levels were made using the Wilcoxon signed-rank test, Kendall’s tau correlation analysis. We divided the lactate levels into intervals based on the interquartile range, and conducted a reliability analysis using using Cohen’s kappa to evaluate agreement between arterial, CSF, and JB lactate levels. All statistical analyses were performed using the PASW-SPSS software version 18 (IBM, Armonk, NY) and MedCalc 15.2.2 (MedCalc Software, Mariakerke, Belgium). Results were considered statistically significant at *p* < 0.05 (two-tailed).

## Results

### Patient characteristics

**Figure 2:**
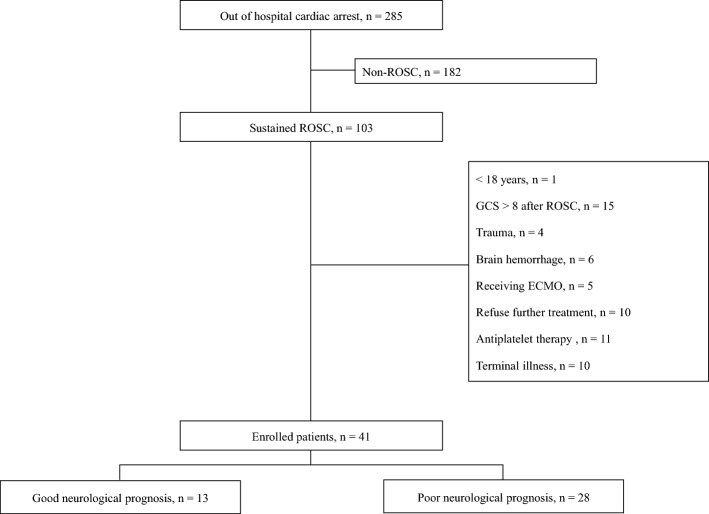
Flowchart of the study. *ROSC* Return of spontaneous circulation, *GCS* Glasgow Coma Scale, *ECMO* Extracorporeal membrane oxygenation.

Of the 41 patients enrolled, 28 (68.3%) had poor neurological prognosis. Among the enrolled patients, 8 (19.5%), 5 (12.2%), 1 (2.4%), 2 (4.9%), and 25 (61.0%) had CPC values of 1, 2, 3, 4, and 5, respectively. Overall, 25 patients had a CPC score of 5; however, among them, 11 died during the study period, 2 died due to pneumonia, and organ donation was performed after brain death in 9 patients (Fig. 2). Regarding patients with witnessed CA and cardiac etiology, a higher number had a good neurological prognosis, whereas a longer low-flow time was associated with a poor neurological prognosis. In contrast-enhanced magnetic resonance imaging, 25 patients exhibited BBB disruption, and 23 (82.1%) out of 28 patients with a poor neurological prognosis showed BBB disruption (Table 1).

**Table 1:** General characteristics.

Variables		Total (n = 41)	Good (n = 13)	Poor (n = 28)	*P* value
Age (years)		58.29 ± 16.38	60.23 ± 11.33	57.39 ± 18.38	0.55
Sex, n (%)	Male	30 (73.2)	12 (92.3)	18 (64.3)	0.06
Witness, n (%)	Yes	24 (58.5)	11 (84.6)	13 (46.4)	0.02
Bystander CPR, n (%)	Yes	28 (68.3)	10 (76.9)	18 (64.3)	0.42
Shockable rhythm, n (%)	Yes	8 (19.5)	4 (30.8)	4 (14.3)	0.22
Cardiac etiology, n (%)	Yes	18 (43.9)	9 (69.2)	9 (32.1)	0.03
No flow time (min)		2.00 (0.00–13.50)	1.00 (0.00–3.00)	9.00 (0.00–19.50)	0.07
Low flow time (min)		25.50 (15.00–38.25)	15.00 (9.50–23.50)	28.00 (19.75–42.75)	0.01
GCS		3 (3.0–3.0)	3 (3.0–3.5)	3 (3.0–3.0)	0.89
BBB disruption	Yes	25 (61.0)	2 (15.4)	23 (82.1)	< 0.001
Serum NSE_72_		60.00 (16.65–203.00)	16.40 (13.05–26.55)	108.20 (49.05–254.75)	< 0.001
CCI		2.00 (1.00–4.00)	3.00 (1.00–4.00)	2.00 (0.25–4.00)	0.88
Hypertension		16 (39.0)	5 (38.5)	11 (39.3)	
Diabetic mellitus		13 (31.7)	6 (46.2)	7 (25.0)	
Myocardial infarction		5 (12.2)	2 (15.4)	3 (10.7)	
Atrial fibrillation		1 (2.4)	1 (7.7)	0 (0.0)	
Lung disease		4 (9.8)	0 (0.0)	4 (14.3)	
Renal disease		8 (19.5)	3 (23.1)	5 (17.9)	

Values are presented as number (%) for categorical variables and mean ± standard deviation or median (interquartile range) for continuous variables depending on normal distribution. A good neurological prognosis was defined as CPC 1 and 2, while a poor neurological prognosis was defined as CPC 3–5.

*CPR* Cardiopulmonary resuscitation, *GCS* Glasgow Coma Scale, *BBB* Blood–brain barrier, *NSE*_*72*_ Neuron specific enolase measured at 72 h after return of spontaneous circulation, *CCI* Charlson comorbidity index.

### Comparison between arterial, JB, and CSF lactate levels

In patients with a good neurological prognosis, CSF lactate levels measured immediately, at 24 h, 48 h, and 72 h after ROSC were 3.40 (2.55–5.25), 2.20 (1.90–4.30), 2.30 (1.65–3.70), 2.10 (1.70–2.85), whereas JB lactate levels measured immediately, at 24 h, 48 h, and 72 h after ROSC were 2.20 (1.80–3.15), 1.30 (0.95–2.25), 1.30 (0.70–1.65), 1.20 (0.70–1.65), (*p* = 0.002, 0.02, 0.001, and 0.02, respectively). In patients with a poor neurological prognosis, CSF lactate levels measured immediately, at 24 h, 48 h, and 72 h after ROSC were 6.15 (4.23–7.70), 4.15 (3.50–5.60), 3.65 (2.60–6.55), 3.15 (2.60–5.95), whereas JB lactate levels measured immediately, at 24 h, 48 h, and 72 h after ROSC were 3.65 (2.13–5.40), 2.20 (1.63–4.38), 1.90 (1.33–3.20), 1.50 (1.30–1.98), (*p* = 0.009, < 0.001, < 0.001, and < 0.001, respectively) (Table 2).

**Table 2:** Comparison between arterial, JB, and CSF lactate levels, with respect to neurological prognosis.

JB and CSF lactate (mmol/L)	Overall			Good (n = 13)			Poor (n = 28)		
Time	JB	CSF	*P* value	JB	CSF	*P* value	JB	CSF	*P* value
Immediately after ROSC	3.00 (1.95–4.85)	5.30 (3.35–6.80)	< 0.001	2.20 (1.80–3.15)	3.40 (2.55–5.25)	0.002	3.65 (2.13–5.40)	6.15 (4.23–7.70)	0.01
24 h after ROSC	2.00 (1.30–2.95)	4.00 (2.45–5.15)	< 0.001	1.30 (0.95–2.25)	2.20 (1.90–4.30)	0.02	2.20 (1.63–4.38)	4.15 (3.50–5.60)	< 0.001
48 h after ROSC	1.60 (1.10–2.15)	3.40 (2.30–5.60)	< 0.001	1.30 (0.70–1.65)	2.30 (1.65–3.70)	0.001	1.90 (1.33–3.20)	3.65 (2.60–6.55)	< 0.001
72 h after ROSC	1.40 (1.15–1.90)	2.90 (2.15–5.70)	< 0.001	1.20 (0.70–1.65)	2.10 (1.70–2.85)	0.02	1.50 (1.30–1.98)	3.15 (2.60–5.95)	< 0.001

Arterial and JB lactate (mmol/L)	Overall			Good (n = 13)			Poor (n = 28)		
Time	Arterial	JB	*P* value	Arterial	JB	*P* value	Arterial	JB	*P* value
Immediately after ROSC	3.30 (2.10–5.00)	3.00 (1.95–4.85)	0.48	2.10 (1.65–2.65)	2.20 (1.80–3.15)	0.25	4.00 (2.93–7.63)	3.65 (2.13–5.40)	0.15
24 h after ROSC	1.90 (1.25–3.40)	2.00 (1.30–2.95)	0.54	1.20 (0.95–1.65)	1.30 (0.95–2.25)	0.42	2.30 (1.60–4.63)	2.20 (1.63–4.38)	0.25
48 h after ROSC	1.60 (1.15–2.20)	1.60 (1.10–2.15)	0.24	1.30 (0.80–1.75)	1.30 (0.70–1.65)	0.23	1.95 (1.43–3.20)	1.90 (1.33–3.20)	0.48
72 h after ROSC	1.60 (1.20–2.15)	1.40 (1.15–1.90)	0.20	0.90 (0.75–1.75)	1.20 (0.70–1.65)	0.35	1.80 (1.33–2.20)	1.50 (1.30–1.98)	0.02

Arterial and CSF lactate (mmol/L)	Overall			Good (n = 13)			Poor (n = 28)		
Time	Arterial	CSF	*P* value	Arterial	CSF	*P* value	Arterial	CSF	*P* value
Immediately after ROSC	3.30 (2.10–5.00)	5.30 (3.35–6.80)	0.003	2.10 (1.65–2.65)	3.40 (2.55–5.25)	0.002	4.00 (2.93–7.63)	6.15 (4.23–7.70)	0.10
24 h after ROSC	1.90 (1.25–3.40)	4.00 (2.45–5.15)	< 0.001	1.20 (0.95–1.65)	2.20 (1.90–4.30)	0.01	2.30 (1.60–4.63)	4.15 (3.50–5.60)	0.001
48 h after ROSC	1.60 (1.15–2.20)	3.40 (2.30–5.60)	< 0.001	1.30 (0.80–1.75)	2.30 (1.65–3.70)	0.002	1.95 (1.43–3.20)	3.65 (2.60–6.55)	< 0.001
72 h after ROSC	1.60 (1.20–2.15)	2.90 (2.15–5.70)	< 0.001	0.90 (0.75–1.75)	2.10 (1.70–2.85)	0.003	1.80 (1.33–2.20)	3.15 (2.60–5.95)	< 0.001

Data are presented as median values (interquartile ranges).

*CSF* Cerebrospinal fluid, *JB* Jugular bulb.

### Correlation between JB lactate levels and CSF lactate levels

**Figure 3:**
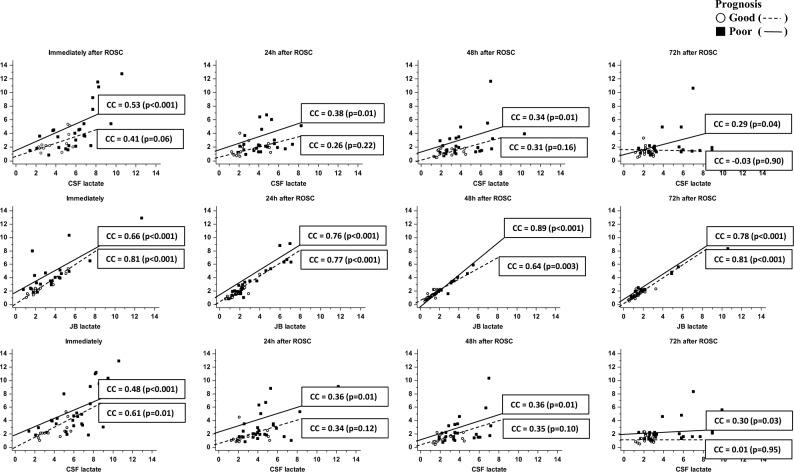
Correlation between jugular bulb (JB) lactate levels and cerebrospinal fluid (CSF) lactate levels, in the context of neurological prognosis, is as follows: For patients with a good neurological prognosis, there were no significant correlations between JB and CSF lactate levels measured immediately, at 24 h, 48 h, and 72 h after ROSC. However, in patients with a poor neurological prognosis, significant correlations between JB and CSF lactate levels measured immediately, at 24 h, 48 h, and 72 h after ROSC were observed. *ROSC* Return of spontaneous circulation, *CC* Correlation coefficient, *JB* Jugular bulb, *CSF* Cerebrospinal fluid.

In patients with a good neurological prognosis, the correlations coefficient between JB and CSF lactate levels measured immediately, at 24 h, 48 h, and 72 h after ROSC were 0.41, 0.26, 0.31, − 0.03 (*p* = 0.06, 0.22, 0.16, and 0.90, respectively). However, in patients with a poor neurological prognosis, the correlations coefficient between JB and CSF lactate levels measured immediately, at 24 h, 48 h, and 72 h after ROSC were 0.53, 0.38, 0.34, 0.29 (*p* < 0.001, 0.01, 0.01, and 0.04, respectively) (Fig. 3).

### The agreement between arterial, JB, and CSF lactate levels

In patients with a good neurological prognosis, kappa between JB and CSF lactate levels measured immediately, at 24 h, 48 h, and 72 h after ROSC were 0.08, 0.36, 0.14, − 0.05 (*p* = 0.65, 0.06, 0.48, and 0.75, respectively). However, in patients with a poor neurological prognosis, kappa between JB and CSF lactate levels measured immediately, at 24 h, 48 h, and 72 h after ROSC were 0.38, 0.21, 0.22, 0.12 (*p* = 0.001, 0.04, 0.04, and 0.27, respectively). In patients with a good neurological prognosis, kappa between JB and arterial lactate levels measured immediately, at 24 h, 48 h, and 72 h after ROSC were 0.33, 0.88, 0.64, 0.54 (*p* = 0.04, < 0.001, 0.001, and 0.001, respectively) (Table 3). In patients with a poor neurological prognosis, kappa between JB and arterial lactate levels measured immediately, at 24 h, 48 h, and 72 h after ROSC were 0.42, 0.66, 0.85, 0.52 (*p* < 0.001, < 0.001, < 0.001, and < 0.001, respectively) (Table 3).

**Table 3:** Agreement between arterial, JB, and CSF lactate levels, in relation to neurological prognosis.

JB and CSF lactate	Overall		Good		Poor	
Time	Kappa	*P* value	Kappa	*P* value	Kappa	*P* value
Immediately after ROSC	0.32	< 0.001	0.08	0.65	0.38	0.001
24 h after ROSC	0.32	< 0.001	0.36	0.06	0.21	0.04
48 h after ROSC	0.25	0.01	0.14	0.48	0.22	0.04
72 h after ROSC	0.12	0.18	− 0.05	0.75	0.12	0.27

Arterial and JB lactate	Overall		Good		Poor	
Time	Kappa	*P* value	Kappa	*P* value	Kappa	*P* value
Immediately after ROSC	0.42	< 0.001	0.33	0.04	0.42	< 0.001
24 h after ROSC	0.74	< 0.001	0.88	< 0.001	0.66	< 0.001
48 h after ROSC	0.81	< 0.001	0.64	0.001	0.85	< 0.001
72 h after ROSC	0.55	< 0.001	0.54	0.001	0.52	< 0.001

Arterial and CSF lactate	Overall		Good		Poor	
Time	Kappa	*P* value	Kappa	*P* value	Kappa	*P* value
Immediately after ROSC	0.42	< 0.001	0.50	0.01	0.31	0.01
24 h after ROSC	0.31	0.001	0.49	0.05	0.15	0.21
48 h after ROSC	0.29	0.001	0.39	0.05	0.18	0.10
72 h after ROSC	0.12	0.18	0.04	0.83	0.07	0.55

*CSF* Cerebrospinal fluid, *JB* Jugular bulb.

## Discussion

In this study, CSF lactate levels were higher than JB lactate levels, and a statistically significant agreement was observed between CSF and JB lactate levels in patients with a poor neurological prognosis compared to those with a good neurological prognosis. Based on the small number of patients with a good neurological prognosis, it remains difficult to make strong statements regarding the relationship observed between JB lactate and CSF lactate levels in patients with a poor neurological prognosis compared to those with a good neurological prognosis (supple 1). In South Korea, euthanasia is not recognized, and even with a CPC score of 5, life-sustaining treatment is continued if consent for the withdrawal of life-sustaining treatment is not granted by the next of kin. Of the 25 patients with CPC scores of 5, 11 died during the study period.

Lactate is slowly transported across the BBB via passive transport. Therefore, CSF lactate reflects the anaerobic metabolism of cerebral glycolysis, independent of serum lactate^10^. Previous studies have demonstrated that the severity of BBB disruption correlates with a poor neurological prognosis in patients with OHCA, and the prognostic performance of serum NSE varies based on BBB disruption^13–15^. In this study using contrast-enhanced magnetic resonance imaging to evaluate BBB disruption, 23 (82.1%) out of 28 patients with poor neurological prognosis showed BBB disruption, and it can be inferred that the agreement between CSF lactate and JB lactate in patients with poor outcomes may be related to BBB disruption. However, further investigation is required to substantiate this hypothesis. Additionally, JB lactate levels measured immediately, at 24 h, 48 h, and 72 h after ROSC exhibited agreement with arterial lactate levels, irrespective of the neurological prognosis. This could be attributed to the JB catheter's inability to reach the lateral venous sinus, where it meets the jugular fossa. This limitation may pose drawbacks for brain metabolism monitoring using JB catheters. Therefore, utilizing JB catheters for brain metabolism monitoring in OHCA patients may have its limitations.

Serum lactate levels tended to increase immediately after ROSC and gradually decreased over time^5,16^. In this study, even among patients with a poor neurological prognosis, there was no agreement between JB and CSF lactate levels measured at 72 h after ROSC, indicating a decrease in serum lactate levels over time following ROSC in patients with OHCA.

In patients with OHCA, lactate levels can increase; however, the mechanisms underlying cerebral ischemia and mitochondrial dysfunction differ. Cerebral ischemia occurs due to a relative insufficiency in the supply of oxygen and glucose compared to cerebral demands. In contrast, mitochondrial dysfunction results in an increase in lactate levels despite a sufficient oxygen and glucose supply^17–21^. Cerebral ischemia and reperfusion-induced mitochondrial dysfunction lead to cellular apoptosis, yet its mechanism is highly complex. Impairment in endoplasmic reticulum-mitochondrial calcium transfer is a primary cause. Reactive oxygen species, free radicals, and inflammatory mediators contribute to mitochondrial damage, resulting in decreased cellular respiration and ultimately leading to cellular apoptosis^22–25^.

Although this was an animal study, treatments such as nitrite therapy, which differ from ischemia-related interventions, are effective in treating mitochondrial dysfunction^26,27^. Nonetheless, several clinical studies^5,6^ have reported that steroid therapy does not improve neurological prognosis in patients with OHCA. In a study investigating the impact of mean arterial blood pressure regulation on the neurological prognosis of OHCA patients through JB based brain metabolism monitoring, lactate/pyruvate ratio exceeding 16 was defined as ischemia when pyruvate is below 70 μM, and exceeding 70 μM was defined as mitochondrial dysfunction. The study reported that mean arterial blood pressure regulation does not improve neurological prognosis in patients with OHCA^28^. In this study, no significant agreement was observed between CSF and JB lactate levels in patients with a good neurological prognosis. Consequently, when neurological prognostic indicators suggest that patients with OHCA are likely to have a good neurological prognosis through several scoring systems, imaging modalities, and biomarkers used to predict the neurological prognosis of patients with OHCA^29–31^, considering the limitations of differentiating between cerebral ischemia and mitochondrial dysfunction using JB lactate is crucial.

This study had several limitations. First, this was a single-center study with a small sample size. Second, we did not continuously assess JB and CSF lactate levels. However, JB and CSF samples were obtained using inserted catheters, and there was no time difference in the collection of these samples. Finally, the parameters were measured until 72 h after ROSC; thus, changes in the parameters over a long duration could not be determined. Further studies are necessary to overcome these limitations.

## Conclusions

This study demonstrated that JB lactate levels exhibited significant agreement with arterial lactate levels, compared to CSF lactate levels. Therefore, this should be considered when using JB lactate to monitor cerebral metabolism.

### Supplementary Information

Supplementary Information.

## Data Availability

Data is provided within the manuscript or supplementary information files.
