# Supplementary material for: The agreement between jugular bulb and cerebrospinal fluid lactate levels in patients with out-of-hospital cardiac arrest
Source: Sci Rep. 2024 Apr 22;14:9219. doi: 10.1038/s41598-024-59986-5 (PMC11035618; doi:10.1038/s41598-024-59986-5)
Supplement: Supplementary file 1 — Supplementary Information. [file 41598_2024_59986_MOESM1_ESM.docx]

Supplement 1. The linear relationship between arterial, JB, and CSF lactate levels, in relation to neurological prognosis

| JB and CSF lactate | Overall | | Good | | Poor | |
| --- | --- | --- | --- | --- | --- | --- |
| Time | Beta | P value | Beta | P value | Beta | P value |
| Immediately after ROSC | 0.54 | < 0.001 | 0.59 | 0.03 | 0.47 | < 0.001 |
| 24 h after ROSC | 0.84 | < 0.001 | 0.33 | 0.45 | 0.80 | < 0.001 |
| 48 h after ROSC | 0.50 | 0.01 | 0.70 | 0.32 | 0.36 | 0.01 |
| 72 h after ROSC | 0.55 | 0.01 | -0.07 | 0.76 | 0.47 | 0.04 |
| Arterial and JB lactate | Overall | | Good | | Poor | |
| Time | Beta | P value | Beta | P value | Beta | P value |
| Immediately after ROSC | 0.92 | < 0.001 | 0.88 | < 0.001 | 0.87 | < 0.001 |
| 24 h after ROSC | 0.95 | < 0.001 | 0.92 | < 0.001 | 0.92 | < 0.001 |
| 48 h after ROSC | 0.90 | < 0.001 | 0.90 | 0.002 | 0.90 | < 0.001 |
| 72 h after ROSC | 0.80 | < 0.001 | 0.66 | < 0.001 | 0.79 | < 0.001 |
| Arterial and CSF lactate | Overall | | Good | | Poor | |
| Time | Beta | P value | Beta | P value | Beta | P value |
| Immediately after ROSC | 0.99 | < 0.001 | 0.72 | 0.02 | 0.49 | < 0.001 |
| 24 h after ROSC | 0.54 | 0.001 | 0.18 | 0.67 | 0.64 | 0.001 |
| 48 h after ROSC | 0.34 | 0.003 | 0.72 | 0.22 | 0.45 | 0.04 |
| 72 h after ROSC | 0.33 | 0.001 | 0.03 | 0.92 | 0.66 | 0.02 |

CSF, cerebrospinal fluid; JB, jugular bulb
